# Weathering Patterns of Ignitable Liquids with the Advanced Distillation Curve Method

**DOI:** 10.6028/jres.118.003

**Published:** 2013-01-17

**Authors:** Thomas J Bruno, Samuel Allen

**Affiliations:** National Institute of Standards and Technology, Boulder, CO 80305

**Keywords:** accelerants, advanced distillation curve, evaporation patterns, ignitable liquids, trace analysis, weathering

## Abstract

One can take advantage of the striking similarity of ignitable liquid vaporization (or weathering) patterns and the separation observed during distillation to predict the composition of residual compounds in fire debris. This is done with the advanced distillation curve (ADC) metrology, which separates a complex fluid by distillation into fractions that are sampled, and for which thermodynamically consistent temperatures are measured at atmospheric pressure. The collected sample fractions can be analyzed by any method that is appropriate. Analytical methods we have applied include gas chromatography (with flame ionization, mass spectrometric and sulfur chemiluminescence detection), thin layer chromatography, FTIR, Karl Fischer coulombic titrimetry, refractometry, corrosivity analysis, neutron activation analysis and cold neutron prompt gamma activation analysis. We have applied this method on product streams such as finished fuels (gasoline, diesel fuels, aviation fuels, rocket propellants), crude oils (including a crude oil made from swine manure) and waste oils streams (used automotive and transformer oils). In this paper, we present results on a variety of ignitable liquids that are not commodity fuels, chosen from the Ignitable Liquids Reference Collection (ILRC). These measurements are assembled into a preliminary database. From this selection, we discuss the significance and forensic application of the temperature data grid and the composition explicit data channel of the ADC.

## 1. Introduction

Fires are responsible for the loss of approximately $6 billion annually in the United States, and approximately $2 billion of this total is due to arson fires. Moreover, each year approximately 500 people die in the U.S. in arson fires [1]. The investigation of arson fires results in a surprisingly low arrest rate (approximately 19 %), and a very low conviction rate (approximately 2 %). On the other hand, there is reason to think that many past convictions for arson and arson related homicides are in fact unjustified [2,3]. The main reason for these problems is the absence of a clear profile of a typical arsonist, but the difficulty inherent in the chemical analysis of fire debris for residual accelerant (or the more modern term, ignitable liquid) is a contributing factor as well.

Many ignitable liquids can be used to start an arson fire, the most common being gasoline, kerosene, charcoal lighter fluid, paint thinners and solvents, and other less common fuels [4,5]. Attention is even being paid to the new alternative fuels such as biodiesel fuel as potential ignitable liquids [6]. Forensic scientists and criminalists must routinely identify and characterize the accelerant or ignitable liquid in a credible, defensible manner. The analysis of fire debris for the presence of residual ignitable liquid has long been an accepted and routine aspect of arson investigations. The techniques available for such analyses have evolved dramatically in recent years. The application of sophisticated techniques, such as nuclear magnetic resonance spectroscopy (^1^H and ^13^C), fluorescence spectroscopy, second derivative ultraviolet spectroscopy, as well as gas and liquid chromatographic techniques, have been used [7–9]. The nature of ignitable liquids as multi-component, moderately volatile fluids makes the technique of gas chromatography the most important and widely used method for fire debris analysis [10,11]. Indeed, the majority of liquid residue analyses done in forensic labs utilize gas chromatography with some combination of detectors and peripherals [4,5,12–19]. The most common is gas chromatography with mass spectrometry as the detector [20–24].

## 2. Ignitable Liquids

Gas chromatographic analyses of as-purchased ignitable liquids often produce chromatograms that are characteristic of each class of fluids. For example, gasolines and kerosenes have familiar elution-intensity characteristics that can be identified by simple pattern recognition methods [23,25–30]. A typical gas chromatogram of a fresh sample of gasoline will show a pattern of isoalkanes in the early elution region, along with some straight chain aliphatics. Later in the chromatogram (in the elution region of seven carbons and higher), one will see a familiar aromatic pattern with substituted ethyl- and dimethyl benzenes. Modern formulations of high antiknock index gasolines are typically high in aromatic content and low in aliphatic content. Naturally, there is a significant variation in gasoline both regionally and seasonally, expanding the variety of potential ignitable liquids that can be at issue in arson investigations. Despite these observations with gasoline, we note that some classes of fluids do not exhibit chromatograms that can be assembled into a neat classification. This will be discussed further below.

**Fig. 1:**
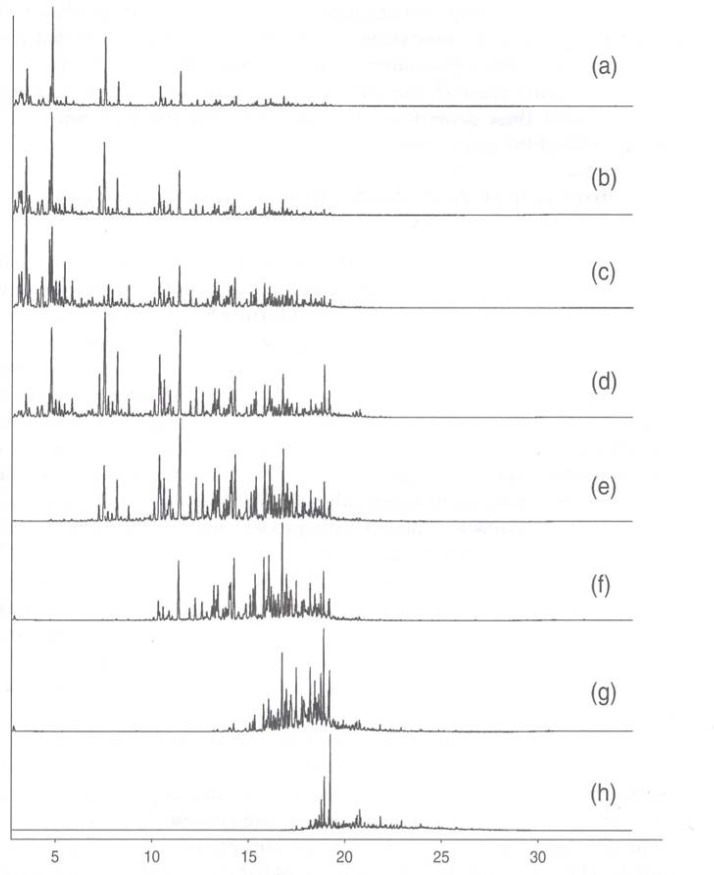
Total ion chromatograms of gasoline at various stages of evaporation. (a) fresh gasoline, (b) 25 % evaporated, (c) 50 % evaporated, (d) 75 % evaporated, (e) 90 % evaporated, (f) 95 % evaporated, (g) 98 % evaporated, (h) gasoline evaporated to dryness. Reproduced with permission from ref. [1].

The National Center for Forensic Science (University of Central Florida) maintains a database (available online) called the Ignitable Liquids Reference Collection (ILRC) [31]. This database is an up to date, comprehensive catalog of ignitable liquids and accompanying characterization data used for the analysis of fire debris samples in accordance established practice, such as those embodied in ASTM practices and procedures. Each entry contains a gas chromatographic profile of the ignitable liquid for which mass spectrometry was used as the detector (gas chromatography – mass spectrometry). This allows the presentation of the total ion chromatogram, and also a description of the predominant ion profile. Moreover, the major peaks are identified, and a link is provided to the NIST Chemistry Web Book for each identified major compound to provide the user with a great deal of additional information [32]. The ILRC categorizes ignitable liquids into useful and distinct families to facilitate searching and reference. These categories, delineated in ASTM method E 1618, include aromatics, gasolines, heavy petroleum distillates, medium petroleum distillates, light petroleum distillates, isoparaffinics, normal alkanes, oxygenates, naphthalenics and miscellaneous compounds. Sources such as this database are invaluable starting points in the analysis of residual ignitable liquid in fire debris. We note, however, that the chromatograms presented in that database are for the as-received, ignitable liquid. The analysis of gasoline (and other ignitable liquids) in fire debris, as presented to the analyst, is far less straightforward than a simple comparison with the chromatogram of the starting fluid. As the sample of gasoline evaporates, the pattern will shift to the later eluting fractions [33]. This is often described as a weathering pattern, clearly illustrated in Fig. 1 for gasoline [11,26]. We note that (to a first approximation) as evaporation of gasoline is continued to dryness, the component suite shifts to heavier, lower vapor pressure, and higher boiling temperature components. Moreover, the suite trends toward enriching the aromatic constituents and depleting the isoalkane constituents. Appreciation of this trend is critical in fire debris analysis, since ignitable liquids have a remarkable persistence when held within the interstices of porous material, even when exposed to elevated temperatures [34–36].

We should add that the weathering pattern developed by evaporation might be different from what may be observed in fire debris because of the potential of intermolecular interactions with the substrate. Thus, we refer to the component suite above as being a first approximation. The typical substrate encountered in fire debris analysis is carpet, carpet padding, or subfloor (made of wood). As the ignitable liquid vaporizes, it is possible for partitioning to take place with the substrate; interactions with the substrate may cause some molecules of the ignitable liquid to be retained more strongly than others. Examination of enthalpy of adsorption measurements of n-alkanes (the most extensively studied family of compounds) shows that many surfaces will interact strongly [37]. While the effect of intermolecular interaction has not been fully studied for typical arson substrates, we point out that the interaction is a surface phenomenon. Thus, we expect the overall effect to be minor. The effect of evaporative weathering will be far more important in affecting the character of residual ignitable liquids in fire debris. Another factor that would cause a difference in the weathering pattern developed by evaporation would be the strong potential of thermal decomposition of the ignitable liquid, a topic treated in more detail later in the paper.

The pattern shift shown in Fig. 1, while specifically for gasoline, is similar in character to the weathering pattern shown by any ignitable liquid that is composed of many components. Clearly, the specific details will differ in terms of the composition suite that will be recovered after progressive evaporation, but the evaporative trend will always be noted. It is interesting that the evaporative trend of the sample is similar to what can be expected upon distillation of the starting fluid. In both cases, the fluid is fractionated primarily by its boiling temperature, and to a lesser extent by the influence of specific intermolecular interactions. Strictly speaking, what is of most interest in fire debris analysis is not the distillate composition, but the composition of what is left in the distillation flask or kettle at a given temperature. These compositions are related, however, through the equation of state of the fluid. The phase diagram provides the four piece puzzle: dew point temperature, dew point composition, bubble point temperature and bubble point composition. Thus, the distillate composition anticipates the residual kettle composition.

## 3. The Advanced Distillation Curve Approach

What is indeed remarkable is the similarity of the evaporation profile of Fig. 1 with the output of a recently introduced technique called the advanced distillation curve (ADC) method [11,38–45]. The method has mainly been used in the characterization of fuels. In general terms, the classical distillation curve of a fluid is a graphical depiction of the boiling temperature of the fluid mixture plotted against the volume fraction distilled. This volume fraction is usually expressed as a cumulative percent of the total volume. One most often thinks of distillation curves in the context of petrochemicals and petroleum refining, but such curves are of great value in assessing the properties of any complex mixture. Indeed, the measurement of distillation curves has been part of complex fluid specifications for a century (typically listed as the fluid volatility), and they are inherent in the design of all fuels.

The ADC, which was developed to improve the classical measurement, is an analytical protocol that can be applied to any complex fluid. It features (1) a composition explicit data channel for each distillate fraction (for both qualitative and quantitative analysis), (2) temperature measurements that are true thermodynamic state points that can be modeled with an equation of state [46–50], (3) temperature, volume and pressure measurements of low uncertainty suitable for equation of state development, (4) consistency with a century of historical data [51], (5) an assessment of the energy content of each distillate fraction [40], (6) trace chemical analysis of each distillate fraction [52,53], (7) corrosivity assessment of each distillate fraction [54,55]. The first aspect summarized above, the composition explicit channel, essentially anticipates the evaporation profile of Fig. 1, as we have reported previously. The other features of the approach provide additional advantages applicable to the study of ignitable liquids, however.

Relating the evaporation profile to true thermodynamic state points of the distillation curve provides a link to thermodynamic theory. Thus, we are able to model the distillation curve resulting from our metrology with model based on an equation of state. Such thermodynamic model development is simply impossible with the classical approach to distillation curve measurement, or with any of the other techniques that are used to assess fuel volatility (or vapor liquid equilibrium). The application of the ADC to the study of fuels used as arson ignitable liquids thus has the potential of validating analyses, and providing a predictive framework to guide analyses of evaporated or weathered ignitable fluids absorbed in fire debris.

**Fig. 2:**
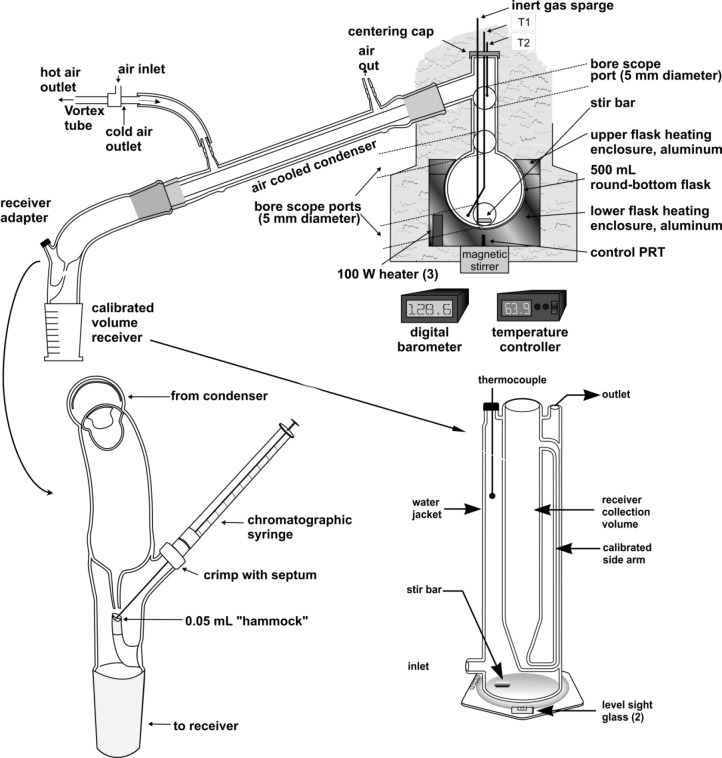
Schematic diagram of the overall apparatus used for the measurement of distillation curves. Expanded views of the sampling adapter and the stabilized receiver are shown in the lower half of the figure.

The apparatus used for the ADC measurement (depicted schematically in Fig. 2) has been described in detail elsewhere, so only a brief summary will be provided here. The distillation flask (in which the fuel sample is placed) is a 500 mL round bottom flask that is inserted in a two-part aluminum heating jacket, the lower part of which is contoured to fit the flask. Cartridge heaters are placed in the lower, contoured part of the jacket. The jacket and heaters are capable of operation up to 350 °C, with a local uniformity of 0.2 °C. Temperature is controlled with a PID controller that is programmed to simulate the fluid behavior. This is called a model predictive temperature controller. The jacket exterior is insulated with a Pyrex wool enclosure.

Three observation ports are provided in the insulation to allow penetration with a flexible, illuminated bore scope. The bore scope ports, illustrated in Fig. 2, are placed to observe the fluid in the boiling flask, the top of the boiling flask (where the spherical section joins the head, and the distillation head (at the bottom of the take-off). Above the distillation flask, a centering adapter provides access for two thermally-tempered J-type thermocouples that enter the distillation head. One thermocouple (TC1 in Fig. 2) enters the distillation flask and is submerged in the fluid, to monitor the temperature of the bulk fluid. This temperature is referred to as T_k_ (signifying its placement in the kettle). This thermocouple is placed well below the surface of the fluid. The other thermocouple (TC2 in Fig. 2) is centered at the low point of distillate take-off (the typical distillation head placement, as is done in classical distillations. The temperature measured directly in the fluid is a true state point that can be essential for modeling studies and comparison with theory. The temperature measured at the bottom of the distillation head take off point, referred to as T_h_, is needed to compare advanced distillation curve measurements with measurements that have been taken for the last century. Beneath the aluminum jacket, a magnetic stirrer drive is positioned to couple with a magnetic stir bar inside the distillation flask. Rapidly stirring the contents of the distillation flask during the measurement is essential for maintaining horizontal temperature uniformity in the fluid. The thermocouples positioned as stated above provide a rapid response to temperature.

**Fig. 3:**
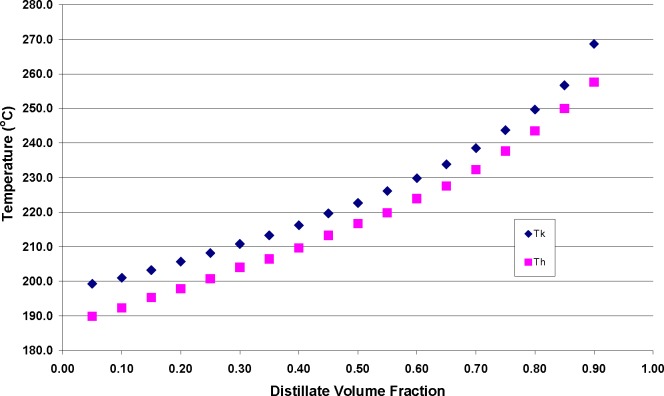
The distillation curve for STP Diesel Fuel Injector Treatment presented in T_k_ and T_h_. The curves presented are the averages of five separate measurements. The uncertainties relating to the measurements have been discussed in the text.

Vaporized fluid taken off the flask is directed into a forced-air condenser chilled with a vortex tube. The vortex tube can produce a cold air stream to a temperature as low as −40 °C. Following the condenser, the distillate enters a newly designed transfer adapter that allows instantaneous sampling of distillate for chemical analysis by any applicable means. The flow path of the distillate is focused to drop into a 0.05 mL “hammock” that is positioned directly below the flow path. A crimp cap fixture is incorporated as a side arm of the adapter, allowing a replaceable silicone or Teflon septum seal) to be positioned in line with the hammock. To sample the distillate, one simply uses a chromatographic syringe equipped with a blunt tipped needle, as shown in Fig. 3.

The composition explicit data channel allows the application of any applicable analytical technique. The most common technique that is applied is gas chromatography with mass spectrometric detection (GC-MS). We have also augmented this with on line FTIR utilizing a light pipe (GC-MS-FTIR), but more commonly we have applied FTIR off-line [55]. Specific element analysis has been applied for sulfur with a sulfur chemiluminescence detector (GC-SCD). The sulfur analysis has been coupled with corrosivity analysis with a small scale copper strip corrosion test [56,57]. We have also applied Karl Fisher coulombic titrimetry and refractometry in specific cases [58], but any analytical method that can be applied to fluid samples can be used.

Our usual practice is to withdraw an aliquot of distillate via syringe, and immediately inject this into a pre-weighed vial containing a suitable solvent. Multiple analyses can subsequently be performed on this vial, and the statistics so generated provide confidence in the result. Moreover, the solvent can often serve to stabilize the sample by minimizing vaporization (with the addition of a keeper solvent) or a preservative. Another advantage of this approach is that the samples of all distillate fractions of a given measurement can be collected and analyzed at one time with an automatic sampler. Of course, it is possible to withdraw the aliquot from the hammock, and inject it immediately into the sampling port of the instrument, but this is rarely done.

When the sample drops from the sampling transfer adapter, it flows into a level stabilized receiver to allow a volume measurement. This receiver consists of a central volume that gradually decreases in diameter at the base, and connects to a smaller-diameter side arm sight glass that is calibrated. The side arm stabilizes the fluid level for a precise volume measurement as the distillation proceeds. The large inner volume and the sight glass are enclosed in a water jacket that contains a thermometer and a magnetic stir bar for circulation. The side arm sight glass allows a volume measurement with an uncertainty of 0.05 mL.

We have applied this metrology to many fluids, most of which are fuels. This work has included the study azeotropes [59], gasolines [41,53,58,60,61], aviation fuels [39,47,50,62–74], diesel fuels [75–85], crude oils [54,55] and rocket propellants [39,86–89]. We note that although the method has multiple features (corrosivity, enthalpy, etc.), it is not always necessary or desirable to apply all of them in every application. The literature that we have developed lends itself naturally to the consideration of ignitable liquids, since many of these fuels have been used in arson. At this time, a comprehensive database of these data does not exist in one depository. For this reason, we list by reference these previous studies as a pointer.

In this paper, we extend the range of ADC application to the visualization of weathering patterns for a collection of ignitable liquids (other than common finished fuels) representing some of the ASTM classes. These classes include aromatics, gasolines, heavy petroleum distillates, medium petroleum distillates, light petroleum distillates, isoparaffinics, normal alkanes, oxygenates, naphthalenic-paraffinics and miscellaneous compounds. For each example we have considered, we measured the thermodynamically consistent distillation curve and a moiety family analysis as a function of distillate volume fraction. We also developed a series of annotated chromatograms (showing the predicted weathering pattern) for each of the ignitable liquids.

## 4. Experimental

Most of the fluids that we measured during the course of this work are listed (by ILRC category) in Table 1 [90]. Each fluid was obtained from either a commercial or military source, and was used either as received, or as withdrawn from its container. Prior to any distillation measurement, the fluid was analyzed by a gas chromatographic method to characterize the fluid and to allow us to optimize the distillation measurement. While each chromatographic method was optimized for the individual sample, all were done with a 30 m capillary column of 5 % phenyl–95 %-dimethyl polysiloxane having a thickness of 1 µm.

The required volume of fluid for the distillation curve measurement (in each case 200 mL) was placed into the boiling flask with a 200 mL volumetric pipette. The thermocouples were then inserted into the proper locations to monitor T_k_, the temperature in the fluid and T_h_, the temperature at the bottom of the take-off position in the distillation head. Enclosure heating was then commenced with a multi-step program based upon our experience with similar fluids, with guidance from the chemical analysis described in the previous paragraph [91]. Volume measurements were made in the level-stabilized receiver, and sample aliquots were collected at the receiver adapter hammock. In the course of this work, we performed between four and six complete distillation curve measurements for each of the fluid samples.

Since the measurements of the distillation curves were performed at ambient atmospheric pressure (measured with an electronic barometer), temperature readings were corrected for what should be obtained at standard atmospheric pressure (1 atm = 101.325 kPa). This adjustment was done with the modified Sydney Young equation, in which the constant term was assigned a value appropriate for the sample [92–95]. This constant is correlated with the average carbon chain length of the fluid being measured, thus the initial chemical analysis of each ignitable liquid is critical in this regard. In some cases, Sydney Young constants that correspond to non-integral carbon numbers were used. The magnitude of the correction is of course dependent upon the extent of departure from standard atmospheric pressure. The location of the laboratory in which the measurements reported herein were performed is approximately 1650 m above sea level, resulting in a typical temperature adjustment of 8 °C. The actual measured temperatures are easily recovered from the Sydney Young equation at each measured atmospheric pressure.

## 5. Results

Brevity dictates that we do not describe in detail the measurements and results on all of the ignitable liquids listed in Table 1; rather we will select and discuss a few representative fluids. These selected examples will allow us to present the salient features of the method that are of particular relevance to the study of ignitable liquids. A complete listing of data and figures covering all the fluids is available as supplementary information.

## 6. ADC Results – Selected Examples

### 6.1 CI Engine Fuel Injector Cleaner

The first ignitable liquid that we will consider is a compression ignition (CI, or diesel) engine fuel injector cleaning fluid. This fluid is sold commercially as an additive to diesel fuel that purports to maintain clean fuel injectors. The particular fluid that we obtained for measurement was sold commercially as STP Diesel Fuel Injector Treatment, classified as a heavy petroleum distillate. The efficacy or performance of this product is irrelevant to our purpose here; we seek merely to examine this fluid in terms of its known potential as an ignitable liquid in arson. This fluid is listed in the ILRC under sample reference No 0014.

We performed five separate distillations for this fluid, primarily to assess the uncertainty in the temperature data grid. During the initial heating of each aliquot in the distillation flask, the behavior of the fluid was carefully observed. Direct observation through the flask window or through the bore scope allowed measurement of the onset of boiling for each of the mixtures (T_k_, measured with TC1 of Fig. 2). Typically, to ascertain the initial boiling behavior, we measure the onset of bubbling, the temperature at which bubbling is sustained, and the temperature at which the vapor rises into the distillation head. We have shown that this last temperature is actually the initial boiling temperature (that is, an approximation of the bubble point temperature at ambient pressure) of the fluid mixture. This measurement is significant for a mixture because it can be modeled with an equation of state. Vapor rise is accompanied by a sharp increase in T_h_ (noted on TC2), and is therefore far less subjective to ascertain and thus is less uncertain than the onset of bubbling. Experience with previous mixtures indicates that the uncertainties in the onset and sustained bubbling temperatures are approximately 1 °C. The expanded uncertainty (considering the repeatability and the calibration) in the vapor rise temperature was 0.3 °C. In Table 2a, we present the initial temperature observations for STP diesel fuel injector treatment. We note that these values are consistent with the classification of this fluid as a heavy petroleum distillate with components ranging up to seventeen carbon atoms.

During the measurement of the distillation curves of this fluid, both the kettle and head temperatures were recorded (T_k_ and T_h_, respectively). The ambient atmospheric pressure was also recorded and used to adjust the temperatures to what would be obtained at sea level atmospheric pressure by use of the modified Sydney Young equation. Each curve was measured five times, and the repeatability in temperature T_k_ was 0.3 °C. The expanded uncertainty of the pressure measurement (considering repeatability and calibration) was 0.002 kPa. The uncertainty in the volume measurement that is used to obtain the distillate volume fraction was 0.05 mL. In Table 2b, we present head and kettle temperatures, as well as the measured atmospheric pressure, as a function of distillate cut. We note a distillation temperature range of approximately 70 °C. This is an appreciable range over which we can expect a significant change in composition. These data are presented graphically in Fig. 3. We note that the distillation proceeds from the initial boiling point and increases to the 90 percent distillate fraction with a sigmoidal-shaped curve.

Clearly, the temperatures of the distillation curve are far lower than combustion temperatures. It is understood, however, that ignitable liquids that are recovered in fire debris (for example, in carpet pad or subfloor) do not encounter combustion temperatures. Rather, they encounter significantly lower temperatures that in fact may not depart very far from the distillation temperatures [34–36]. Otherwise, no recovery of residual fluid from fire debris would ever be possible. When one considers the behavior of ignitable liquids in fire debris analysis, the region of the distillation curve that is of most interest is usually the later part, but for reasons that will become clear later, it is important that the entire curve be available for examination.

We note from Fig. 3 that the temperature, T_k_, exceeds (or leads) T_h_ by approximately 7 °C over the entire curve. This is typical of complex fluids in which azeotropy does not occur. If azeotropic mixtures were present among major constituents, one would observe regions along the curve in which T_k_ and T_h_ converge [59]. Since azeotropy among mixture constituents would cause the components of the mixture to behave like pure fluids, it is critical in the prediction of weathering patterns that such behavior be detected.

While the gross examination of the distillation curves is instructive and valuable for many fluid science purposes, the composition channel of the advanced approach can provide even greater understanding and information content, and this is especially relevant to visualizing the weathering pattern. One can sample and examine the individual fractions as they emerge from the condenser, and relate them to the temperature data grid of Fig. 3. This was done by withdrawing 7 μL aliquots of distillate (as a function of distillate volume fraction) and diluting this in a known mass (approximately 1 mL) of n-hexane. Each of these fractions thus prepared was analyzed by a gas chromatographic mass spectrometric method (30 m capillary column of 5 % phenyl–95 %-dimethyl polysiloxane having a thickness of 1 µm, temperature program from 90 to 275 °C, 9 °C per minute, mass spectrometer set to record an ion mass range of 15 to 550 Da).

**Fig. 4:**
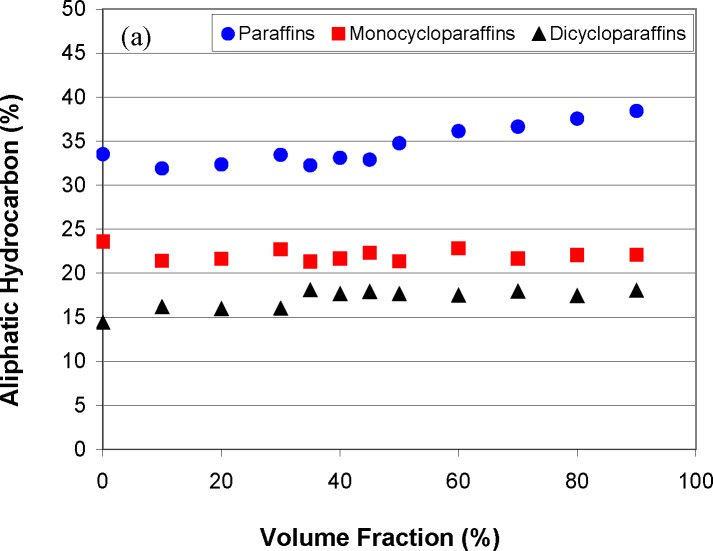

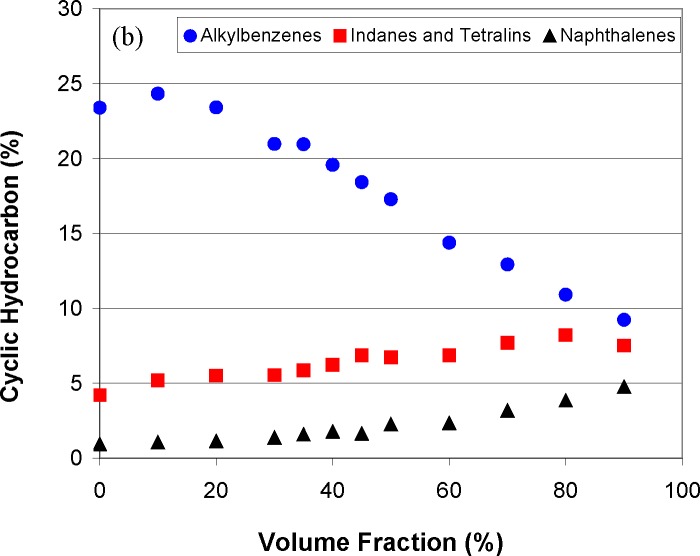
a. A summary of the moiety family analysis for the aliphatic hydrocarbons of STP Diesel Fuel Injector Treatment. b. A summary of the moiety family analysis for the cyclic hydrocarbons of STP Diesel Fuel Injector Treatment.

Before we discuss the detailed chromatographic analysis as a function of distillate volume fraction, we can provide a general overview of the composition by a moiety family analysis method that is based on the ASTM Method D-2789 [96]. In this method, one uses the GC-MS to classify hydrocarbon samples into six different types. The six different moieties are paraffins, monocycloparaffins, dicycloparaffins, alkylbenzenes (or aromatics), indanes and tetralins, and naphthalenes. While strictly applicable to low olefinnic gasolines, it is routinely used for the study of all fuels. The solvent, n-hexane, does not interfere with the main chromatographic peaks, and is not used in the subsequent calculation of moiety families. The results of these analyses (as % volume fractions) are presented in Fig. 4a and 4b. We note from Fig. 4a that the relative concentration of aliphatic constituents are approximately constant through the vaporization, but from Fig. 4b we note a very different behavior for alkylbenzenes, indanes, tetralins and naphthalenes. The alkylbenzene content, in particular, decreased markedly as vaporization proceeds, while there is a slight increase in the content of indanes, tetralins and naphthalenes. The consequence for the prediction of fire debris weathering patterns is clear; one can expect approximately similar aliphatic content in the weathered fluid as in the virgin fluid, albeit shifted to higher relative molecular mass. From Fig. 4b, on the other hand, we are led to expect a weathering pattern with a much lower content of alkylbenzenes, accompanied by a slight increase in indanes, tetralins and naphthalenes.

**Fig. 5:**
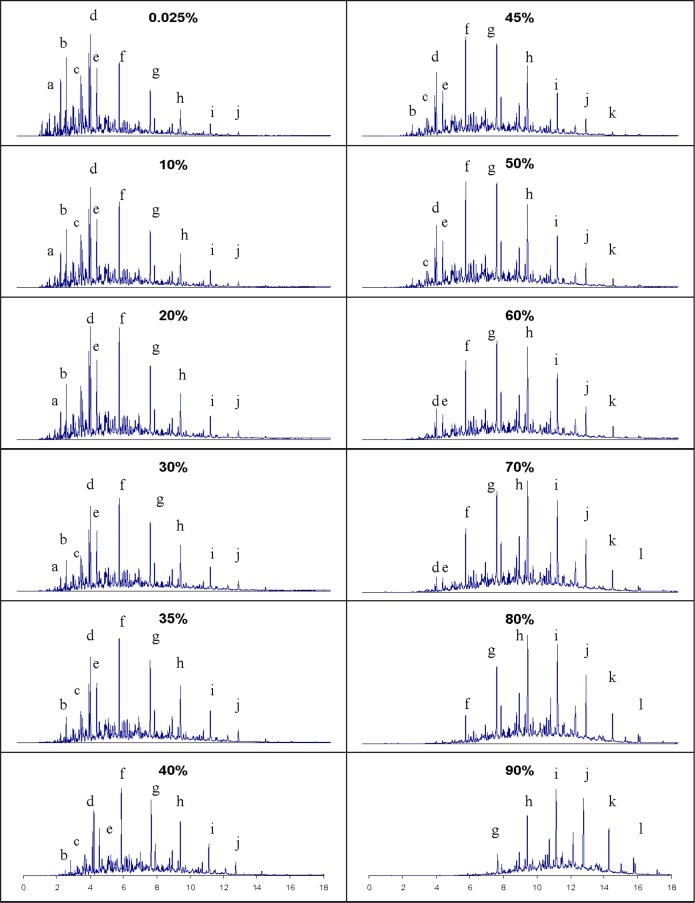
Chromatographic behavior of STP Diesel Fuel Injector Treatment as a function of distillate volume fraction. Identified components are: (a) xylene (b) nonane (c) 1-ethyl-3-methylbenzene (d) decane (e) 1,2,4-trimethylbenzene (f) undecane (g) dodecane (h) tridecane (i) tetradecane (j) pentadecane (k) hexadecane (l) heptadecane.

The general survey provided by the above technique is valuable as a starting point, but the composition explicit data channel of the ADC allows a more detailed analysis for each distillate fraction. This provides greater insight into the weathering pattern that may be expected for an ignitable liquid recovered in fire debris. The samples collected during the distillation can be used for analyses at any desired level of detail. In Fig. 5 we present the total ion chromatograms of the analysis of each fraction, with the major components labeled. The solvent, n-hexane, eluted before the main cluster of components, and was removed electronically. We note how the components progress from light to heavier (generally increasing carbon number) as the distillation proceeds, while in the later stages we note the disappearance of alkylbezene compounds. This is consistent with the moiety family analysis presented above, but with a greater level of detail. What we would expect to find in fire debris is a suite of compounds such as those represented in the 70 to 90 percent distillate fractions. Indeed, this is consistent with the kind of weathering patterns (as in Fig. 1) that are known in ignitable liquids. The advantage of the application of the ADC in this situation is that the information in Figs. 3–5 (assuming one distillation, and sample preparation for the GC-MS analyses) can be obtained in approximately 1.5 hrs rather than the weeks needed for typical weathering studies.

### 6.2 Plastic Adhesive Solvent Promoter

**Fig. 6:**
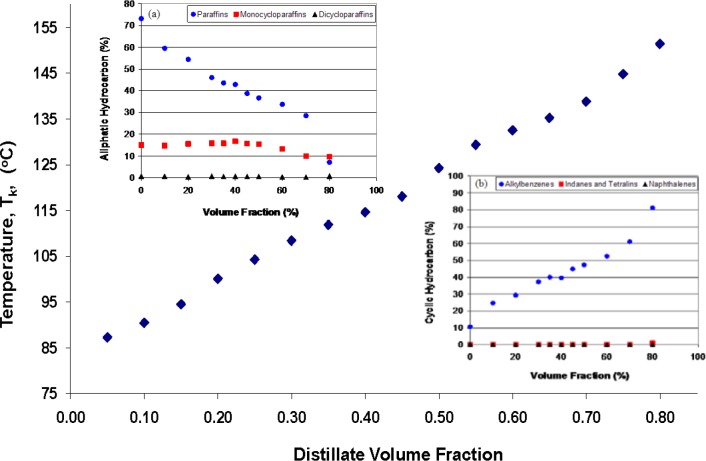
The distillation curve, presented in T_k_, for SEM Plastic Adhesive Promoter, with insets showing the moiety family analysis.

**Fig. 7:**
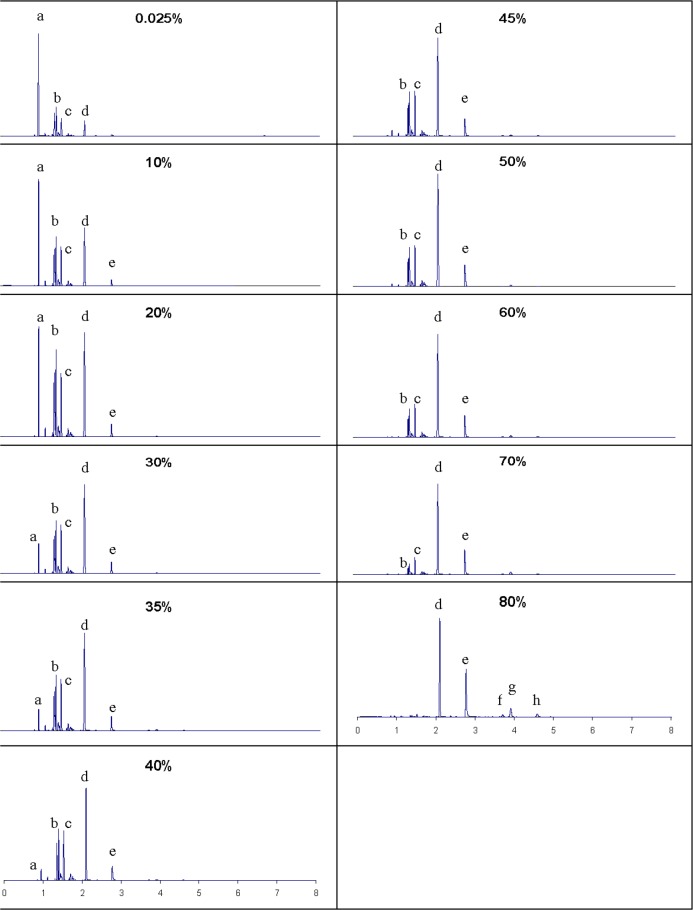
Chromatographic behavior of SEM Plastic Adhesive Promoter as a function of distillate volume fraction. Identified components are: (a) acetone (b) 3-methylhexane (c) heptane (d) toluene (e) butyl acetate (f) ethylbenzene (g) m,p-xylene (h) o-xylene.

The next ignitable liquid we will discuss is SEM Plastic Adhesive Promoter, a solvent classified as a light petroleum distillate that is used to prepare surfaces in preparation for adhesive construction. It is included in the ILRC database under sample reference No 0214. For brevity, we show in Fig. 6 the distillation curve in T_k_ (not presented are the T_h_ data, which when examined with T_k_ do not show azeotropic convergence), with the moiety family analysis shown superimposed as insets. The range of the distillation is approximately 70 °C (as was the case with the previous sample), thus we can expect a significant change in composition to accompany this change. This composition change is clearly reflected in the moiety family analysis, where we observe a decrease in paraffins from a volume fraction of approximately 72 to 28 percent, accompanied by an increase in alkylaromatics from 10 to 80 percent. In Fig. 7, we present the total ion chromatograms of the analysis of each fraction, with the major components labeled. The solvent was again removed electronically. We note how the components progress from light to heavier (generally increasing carbon number) as the distillation proceeds, while in the later stages we note the increase of alkylbezene compounds. This is consistent with the moiety family analysis presented above. Following the same thoughts of our discussion of the previous sample, were this solvent used as an ignitable liquid, we would expect to find in fire debris a suite of compounds such as those represented in the 70 to 90 percent distillate fractions.

### 6.3 Turpentine

**Fig. 8:**
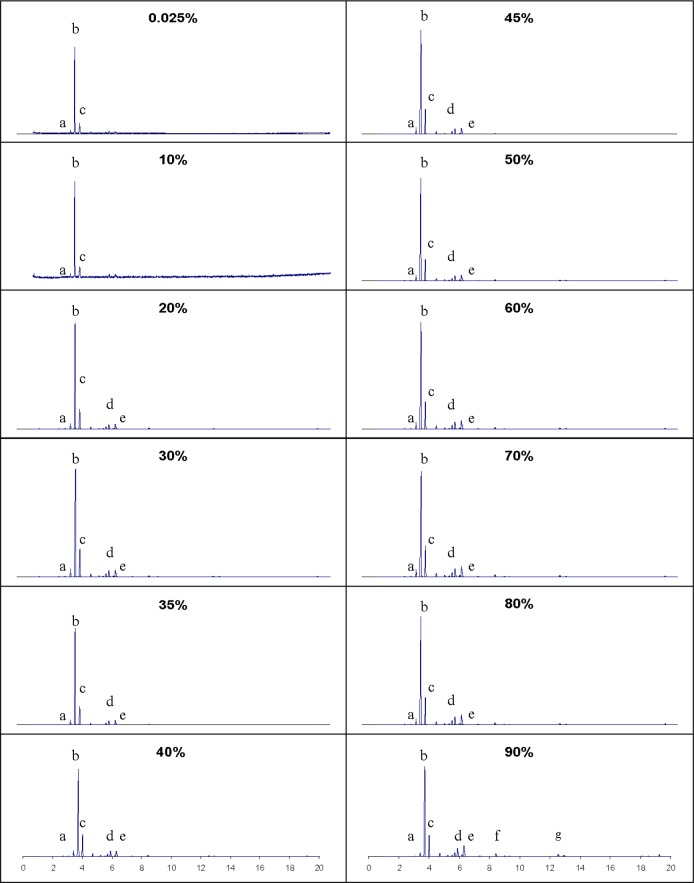
Chromatographic behavior of turpentine as a function of distillate volume fraction. Identified components are: a) tricyclene (b) (−)-α-pinene, (+)-α-pinene racemate, (c) camphene (d) 1,4-cineole (e) limonene (f) terpinolene (g) turpineol.

We mentioned above that the temperature range of the distillation curve is a significant predictor of composition change. Indeed, we have examined many ignitable liquids in which the temperature change is modest (10 °C or less), or in which the temperature is approximately constant over the entire distillation curve. An example of an ignitable liquid that shows a very modest distillation temperature range is industrial turpentine, which typically distills over a range of 10 °C (the details of the distillation measurements performed on this liquid can be found in the supplementary information). Despite the modest distillation temperature range, we nevertheless noted a gradual progression in the composition profile over this temperature range. The components observed are: a) tricyclene, (b) (−)-α-pinene, (+)-α-pinene racemate, (c) camphene, (d) 1,4-cineole (e), limonene, (f) terpinolene, and (g) turpineol. All of these components are present from the 0.025 to the 90 percent distillate volume fraction, but as we can observe in Fig. 8, the clear trend is to enrich in the heavier components (terpinolene and turpineol) while diminishing the lightest component, tricyclene. Despite this trend, the dominant component over the entire vaporization range is the pinene racemate. We also note that the compositional differences between industrial turpentines and the pyrolysis products seen in lumber combustion can be visualized by the composition channel. This is often a source of confusion in fire debris analysis. The ADC can also visualize and predict the differences in compositions known to occur in turpentines made from forest woods in the United States and in Canada [97,98].

### 6.4 SI Engine Fuel Injector Cleaner

This kind of behavior observed above (with ignitable liquids having modest distillation temperature ranges) becomes even more pronounced with some fuel injector cleaners that have only a few components, with the major component (80 %, mass/mass) typically being methanol. In these instances, the distillation curve will be flat, and the composition will, as expected, reflect the composition profile present at the late stage of vaporization, but dominated by methanol.

An example of a fluid that shows a flat distillation curve is Gold Eagle Fuel Injector Cleaner. This is a product that is marketed as a solvent added to gasoline to remove deposits from injectors in spark ignition (SI) engines. This fluid is not specifically listed by name in the ILRC database, although several related fluids are present. We found that this fluid distilled over the entire volume range at a constant T_k_ temperature of approximately 64.5 °C, and the T_h_ temperature of 64 °C. No convergence of T_k_ and T_h_ was noted, a constant displacement of 0.6 °C between the two temperatures. The composition channel of the ADC revealed that the fluid was essentially pure methanol, over the entire distillation curve. For this fluid, the chromatographic profile of the starting material is a good representation of the pattern that could potentially be recovered in fire debris.

## 7. Ignitable Liquid Stability

One complicating factor that must be considered when developing weathering patterns is the potential of chemical reactions. Evidence of thermal decomposition is never visualized in classical evaporation studies, a major drawback. High temperatures can result in cracking of larger molecules into smaller molecules, and also the development of polymers and carbonaceous structures. For example, we have found thermal stress can produce a suite of decomposition products in some fluids, while other fluids are relatively stable. For example, the reactivity of methanol has been noted in numerous studies of the thermophysical properties and in supercritical fluid extraction, where it has been used as an entrainer or co-solvent [99–101]. The composition explicit data channel of the ADC can provide guidance, however, in that often the reaction products due to thermal decomposition are detected. If desired, one can specifically look for potential reaction products, which, in the case of methanol (for example, in the fuel injector cleaner discussed in the prior section) would include dimethyl ether, formaldehyde, acetals and hemiacetals in addition to some hydrogen and carbon monoxide. Clearly, some ignitable liquids are more reactive than others. Linear, branched and aromatic hydrocarbons are stable under the typical conditions of distillation, but the presence of double bonds on the molecular backbone can increase reactivity, and one must be aware of this possibility. An ignitable liquid that has recently come to the fore is biodiesel fuel [6]. Some of the fatty acid ester constituents have one or more double bonds, making them subject to Diels-Alder reactions that result in larger species. In some cases, the reaction products may have to analyzed by liquid chromatographic techniques (HPLC). We note that this is a major advantage of the ADC over even the best classical studies of evaporation weathering. Evaporation studies will not visualize or predict the decomposition products that are associated with thermal stress, the ADC approach will do so, however. Indeed, the stability of biodiesel fuel has been studied directly with the ADC [84,102]. Since the reaction products can be light, with a high vapor pressures (as is the case with thermally stressed methanol), or heavy, with low vapor pressures (as is the case with thermally stressed biodiesel fuel), the entire distillation curve is important.

## 8. Influence of the Substrate

**Fig. 9:**
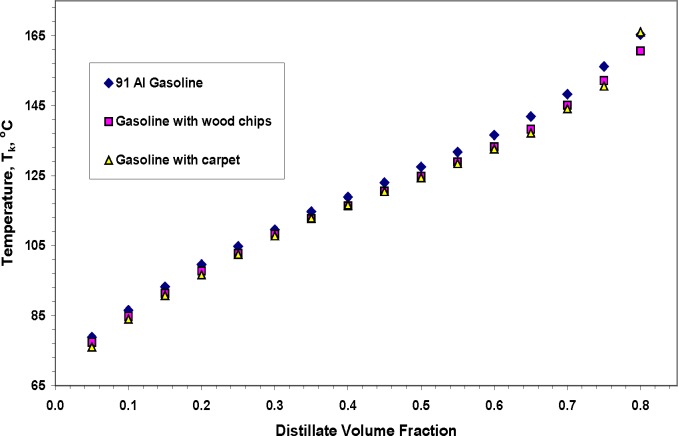
Distillation curves of 91 AI winter quarter gasoline and that gasoline in the presence of wood chips and chopped carpet.

We conclude the description of our results with a preliminary observation regarding the presence of an ignitable liquid on a substrate. In an effort to approximate the effect of the substrate on the temperature data grid of the distillation curve, we performed distillations of the 91 AI summer grade gasoline in the presence of wood chips and coarsely chopped carpet. The wood chips we used were randomly shaped slivers of dried hickory, with a size ranging from 1 – 2 cm on edge, approximately 0.5 cm thick. The carpet with its underlayment was cut with a scissor from a remnant, into squares of approximately 1 cm on edge. Distillations were performed in the usual manner, with 200 mL of the gasoline, into which approximately 3.7 g of either the wood chips or the chopped carpet were added. This resulted in a solids mass fraction of 2.5 %. These quantities of solids do not impede the function of the stirrer, thus no additional uncertainty is introduced into the temperature measurement. Typical distillation curves measured for these tests are provided in Fig. 9, in which we present measurements with a 91 AI gasoline. This fluid was chosen because of its wide boiling temperature range. We note that the major effect of the substrate was to decrease the measured distillation temperatures by between 1 and 3 °C. This lowering appears to be more pronounced with the carpet than with the wood, although the uncertainty of the measurements prevents a definitive conclusion in this regard. It is likely that this observation results from the increased number of nucleation sites available to the gasoline in the presence of the substrate. This allows a faster rate of vaporization for a given heat input (through the aluminum enclosure surrounding the distillation flask).

It is possible that if the heat input (or rather the rate of the temperature program ramping) were made slower, the observation of lower vaporization temperatures would vanish. We chose not to do that experiment, however, since this runs counter to the situation encountered in a fire. The reverse experiment, in which the heating is done at a far more rapid rate, would be instructive. Doing so with the current apparatus would not be possible, however, since the large thermal mass of the enclosure is simply not designed for such rapid heating. We are currently considering designs for an approach that might enable this experiment to be performed. We are also considering modifications to allow a larger solids fraction. We recognize that in this preliminary experiment we have only addressed the temperature data grid in the presence of a substrate. We have not addressed the effect of the substrate on the composition of the fluids recovered in the fire debris. Clearly, any substrate will contribute to the suite of recovered materials, and these compounds must be differentiated from ignitable liquids 16,28,29]. This aspect is currently being addressed with the ADC approach, and will be reported later.

## 9. Relation of Composition Explicit Distillation Data to Fire Debris Analysis

Indeed, the most important aspect of the application of the ADC to the prediction of fire debris analysis profiles lies in the comparison with such analysis. In separate but related work, we have applied PLOT-cryoadsorption to the analysis of fire debris produced on two types of wood with numerous ignitable liquids [103]. PLOT-cryoadsorption is a headspace sampling method [104] that has been applied to the characterization of energetic materials vapors [105], the early detection of food spoilage [106] and the detection of grave soil (that is, illegally buried corpses) [107]. Although a detailed description of the results of fire debris analysis mentioned above is beyond the scope of this paper, we can describe in general terms the similarities between two illustrative examples.

A typical as-received diesel fuel will have an alkylbenzene content of between 18 and 20 %, mass/mass, on the basis of ASTM D-5186 [108]. During an ADC measurement, on will note the sharp drop of alkylbenzenes in the distillate as the temperature increases. From those observations on diesel fuels, cited earlier in the introduction, we measured an alkylbenzene content of between 5 and 6 % percent, and a napthalenic content of approximately 4 %, at the 80 distillate volume fraction. Consequently, one would expect a correspondingly low quantity in fire debris accelerated with diesel fuel. We measured in the headspace (by use of PLOT cryoadsorption coupled with GCMS) of such a fire debris sample 4.9 % (mass/mass) alkylaromatic and 3.2 % (mass/mass) naphthalenic content. The most prevalent compounds recovered from the fire debris were linear and branched alkanes (91.9 %, mass/mass), as predicted by the ADC. The uncertainty in the chromatographic method was 0.2 %, mass/mass. More details on all the fire debris measurements performed with this method will be presented in the future.

## 10. Conclusions

In this paper we have presented examples of the application of the advanced distillation curve method to the characterization of ignitable liquids of relevance to the analysis of fire debris. Moreover, we have provided a database of measurements performed on ignitable liquids other than common finished fuels. This follows our earlier work on numerous motor and aviation fuels; here our focus has been other commercial fluids such as functional additives, lubricants, illumination fuels and solvents. Useful forensic information can be obtained from the temperature data grid (T_k_, T_h_, plotted against distillate volume fraction) and the composition explicit data channel. The range of the distillation temperatures (T_k_) provides an indication of the complexity of the fluid. A large temperature range is typically observed with a more complex fluid in terms of a multiplicity of components. In these situations, one may expect a significant change in composition as the ignitable liquid weathers. Likewise, when the change in temperature is very small, a very simple fluid (a single component or a few close boiling components) is indicated. Of course, there are exceptions to these general guidelines, such as is encountered with a binary mixture of fluids with very different boiling temperatures. These are rarely encountered in arson investigations, however. Another valuable aspect of the temperature data grid is indications of azeotropy potential. The convergence of T_k_ and T_h_ (when plotted together on a distillation curve) occurs when azeotropy is exhibited among constituents of the mixture. Although we have not explicitly demonstrated this with an example, in this paper, our earlier work on motor fuels shows this feature with gasoline that is oxygenated with methanol or ethanol.

The composition channel of data of the ADC is especially useful for forensic applications. We have demonstrated how this aspect allows the weathering patterns for ignitable liquids to be visualized (by applying gas chromatography – mass spectrometry to the later parts of the distillation curve). We have also shown the value of moiety family analyses, and analyses done with specific detectors. The decomposition that one would expect to occur with ignitable liquids during weathering can also be observed explicitly, as we have done with gasoline oxygenates and biodiesel fuel. When considering ignitable liquids that are susceptible to decomposition during thermal stress, the ADC approach has a particular advantage over classical evaporation studies. The ADC will explicitly show the decomposition products, be they heavy or light.

We finish our conclusions with just a few sentences on the thermodynamic modeling aspect that was mentioned in the introduction. The fine details of this work are beyond the scope of this paper; the reader is referred to descriptions of our modeling work for more information. In general terms, our approach is to represent the molar Helmholtz energy, *a*, of a mixture as a sum of an ideal solution contribution and an excess contribution; we can use the theoretical formalism in two different ways. First, we can correlate experimental property data, producing a model to represent the data within experimental uncertainty. Second, we can use the model predictively to estimate property values, based on limited experimental data. With the ADC as a primary experimental input, we have used both of these approaches for aviation fuels, rocket propellants and diesel fuels. Here, it is important to utilize the entire temperature range of the distillation curve in the model development. Once derived, the model affords us the ability to predict distillation (or weathering) temperatures, and in some cases their associated compositions. This aspect is the topic of active research, and will be reported further in the near future.

## Figures and Tables

**Table 1:** A listing of the ignitable liquids studies in this work, arranged according to the classifications in the Reference Collection (ILRC)

Light Petroleum Distillate	Medium Petroleum Distillate	Heavy Petroleum Distillate	Oxygenated	Normal Alkane	Isoparaffinic	Miscellaneous	Aromatic
							
• Klean Strip VM&P Naphtha • CRC Belt Dressing	• WD-40 • E-Z Paint Thinner • Kingsford Odorless Charcoal Lighter • 2-cycle oil and gasoline (16:1)	• STP Gas Treatment • STP Diesel Fuel Injector Cleaner • Interlux Solvent 333 Brushing Liquid • Gunk Liquid Wrench • Gold Eagle Quantum Octane Booster	• Interlux Reducing Solvent 2316N • Interlux Reducing Solvent 2333N	• Tiki Ultra-Pure Lamp Oil • Lamplight Medallion Lamp Oil • Crown Paint Thinner	• Weiman Wax Away	• Klean Strip Pure Gum Spirits Turpentine • Gold Eagle Fuel Injector Cleaner	• 3M General Purpose Adhesive Cleaner • SEM Solve Wax and Grease Remover • Interlux Special Thinner 216 • Norton Adhesive Cleaner

**Table 2a:** The initial boiling temperatures of STP Diesel Fuel Injector Treatment. These temperatures have been adjusted to 1 atm with the Sydney Young equation. The pressures at which the measurements were made are provided for each fuel to permit recovery of the actual measured temperature. The uncertainty (with a coverage factor k=2) in the onset and sustained bubbling temperatures is ~2 °C. The uncertainty in the vapor rise temperature is actually much lower, at ~0.2 °C.

Observed Temperature	STP Diesel Fuel Injector Treatment °C (83.3 kPa)
Onset	159.1
Sustained	189.2
**Vapor Rise**	**197.1**

**Table 2b:** The average measured distillation curve data (that is, the temperature data grid) for STP Diesel Fuel Injector Treatment. These temperatures have been adjusted to 1 atm with the Sydney Young equation; the experimental atmospheric pressures are provided to allow recovery of the actual measured temperatures. The uncertainty in the temperature measurements is 0.3 °C.

Distillate Volume Fraction, %	STP Diesel Fuel Injector Treatment (83.3 kPa)	
	T_k_, °C	T_h_, °C
5	199.3	189.9
10	201.0	192.3
15	203.3	195.3
20	205.7	197.9
25	208.2	200.7
30	210.8	204.0
35	213.3	206.5
40	216.2	209.6
45	219.7	213.3
50	222.7	216.7
55	226.1	219.8
60	229.8	223.9
65	233.8	227.5
70	238.5	232.3
75	243.7	237.7
80	249.7	243.5
85	256.7	250.0
90	268.7	257.6
